# Sarcophine-Diol, a Skin Cancer Chemopreventive Agent, Inhibits Proliferation and Stimulates Apoptosis in Mouse Melanoma B_16_F_10_ Cell Line

**DOI:** 10.3390/md10010001

**Published:** 2011-12-22

**Authors:** Pawel T. Szymanski, Bhimanna Kuppast, Safwat A. Ahmed, Sherief Khalifa, Hesham Fahmy

**Affiliations:** 1 Department of Pharmaceutical Sciences, College of Pharmacy, South Dakota State University, Brookings, SD 57007, USA; Email: pawel.szymanski@sdstate.edu (P.T.S.); bhimanna.kuppast@sdstate.edu (B.K.); 2 Department of Pharmacognosy, Faculty of Pharmacy, Suez Canal University, Ismailia 41522, Egypt; Email: safwat_aa@yahoo.com; 3 College of Pharmacy, Qatar University, Doha 02713, Qatar; Email: sherief@qu.edu.qa

**Keywords:** sarcophine, sarcodiol, chemoprevention, skin cancer, melanoma

## Abstract

Sarcodiol (SD) is a semi-synthetic derivative of sarcophine, a marine natural product. In our previous work, we reported the significant chemopreventive effects of SD against non-melanoma skin cancer both *in vitro* and *in vivo* mouse models. In this investigation, we extended this work to study the effect of sarcodiol on melanoma development, the more deadly form of skin cancer, using the mouse melanoma B_16_F_10_ cell line. In this study we report that SD inhibits the *de novo* DNA synthesis and enhances fragmentation of DNA. We also evaluated the antitumor effect of SD on melanoma cell viability using several biomarkers for cell proliferation and apoptosis. SD inhibits the expression levels of signal transducers and activators of transcription protein (STAT-3) and cyclin D1, an activator of cyclin-dependent kinase 4 (Cdk4). SD treatment also enhances cellular level of tumor suppressor protein 53 (p53) and stimulates cleavage of the nuclear poly (ADP-ribose) polymerase (cleaved-PARP). SD also enhances cellular levels of cleaved Caspase-3, -8, -9 and stimulates enzymatic activities of Caspase-3, -8 and -9. These results, in addition to inhibition of cell viability, suggest that SD inhibits melanoma cell proliferation by arresting the cell-division cycle in a Go quiescent phase and activates programmed cell death (apoptosis) via extrinsic and intrinsic pathways. Finally, these studies demonstrate that SD shows a very promising chemopreventive effect in melanoma B_16_F_10_ tumor cells.

## 1. Introduction

According to the American Academy of Dermatology, one in five Americans will develop some form of skin cancer in their life [1,2,3,4]. Fortunately most skin cancers can be detected in their early stages because skin tumors are more visible than others. Three types of cancer account for virtually 100% of skin cancer cases. The non-melanoma skin cancers, including basal cell carcinoma and squamous cell carcinoma are not lethal and are easily cured [5]. Malignant melanoma is the third and most deadly type of skin cancer causing the majority (about 75%) of deaths related to skin cancer [6].

Melanoma is a cancer that develops in melanocytes, the pigment cells in the skin. It can be more serious than the other types of skin cancer. It may spread to other parts of the body (metastasize) and cause serious illness and death. One historical example lies in 1960s examination of nine Peruvian mummies, radiocarbon dated to be approximately 2400 years old, which showed apparent signs of melanoma: melanoma masses in the skin and diffuse metastases to the bones [7]. It is also worth mentioning that about 70,000 new cases of melanoma cancer were diagnosed in the United States in 2004 [8].

Currently, surgical intervention is the most common form for skin cancer treatment. The goal of excision is to remove tumor, prevent spreading of the cancer and to restore normal function of the skin. The survival rates for patients with melanoma cancer vary, depending on how early the cancer was detected and surgically removed as well as the precision of the tumor removal, which is not always 100% accurate.

Recently, there has been considerable interest in the use of marine natural products for chemopreventive activity against skin cancer development [1,9,10,11,12]. Sarcophytol A, a cembranoid isolated from the Okinawan soft coral *Sarcophyton glaucum*, was reported to have anti-cancer activity, particularly against skin tumors [13,14]. It has already been studied by the National Cancer Institute at a preclinical trial level [1]. However, the major limitation with sarcophytol A is its supply. Sarcophytol A is available only in very small quantities in the soft coral.

**Figure 1:**
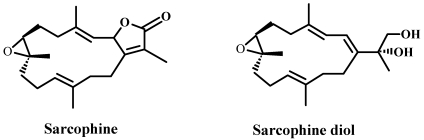
Structures of sarcophine and sarcophine-diol.

For this particular reason, in our laboratory, studies have been carried out on sarcophine. Sarcophine is one of the most abundant cembranolides isolated from the red Sea coral *Sarcophyton glaucum*, with yields up to 3% of animal dry weight [15]. We have reported that structural modifications of sarcophine yielded sarodiol (SD) and sarcotriol (ST). Our studies demonstrated that SD displayed a very promising chemopreventive effect against Non-melanoma skin cancer both *in vitro* and *in vivo* as well as using several biomarkers for cell proliferation and apoptosis. We demonstrated that topical application of SD decreases expression level of cyclooxygenase-2 (Cox-2), a protein marker for inflammation and enhances cellular expression levels of cleaved-Caspase-3 and -8, molecular markers for apoptosis in female CD-1 mice with 7,12-dimethybenz(a)antracene (DMBA) initiated and 12-0-tetradecanoylphorbol-13-acetate (TPA) promoted skin cancer [1]. We also found that SD treatment inhibits skin tumor development (both incidence and multiplicity) in both chemically induced skin cancer in female CD-1 mice and also UVB-induced skin cancer in SKH-1 hairless mice [2]. Topical application of SD also enhances cellular expression levels of cleaved-Caspase-3 and -8, and increases the rates of DNA fragmentation in skin cells isolated these mice [2]. Our subsequent studies demonstrated that SD decreases cell viability in the human epidermoid carcinoma A431 cell line in a concentration-dependent manner [3]. These findings also showed that SD inhibits cell proliferation, as determined by lower levels of incorporation of the thymidine analogue 5-bromo-2′-deoxyuridine (BrdU) onto *de novo* synthesized DNA and simultaneously enhances fragmentation of DNA in the human epidermoid carcinoma A431 cell line [3]. Moreover, these studies showed that SD increases expression cellular expression levels of caspase-3 through activation of upstream caspase-8 in these cancer cells. (Structures of compounds are shown in Figure 1).

In the present investigation, we have extended our previous research to evaluate the effect of sarcodiol on melanoma using mouse melanoma B_16_F_10_ cell line. Our results show that SD inhibits *de novo* DNA synthesis and enhances fragmentation of DNA. We also demonstrate that SD inhibits the expression levels of signal transducer and activator of transcription protein (STAT-3) and cyclin D1, an activator of cyclin-dependent kinase 4 (Cdk4) and markers of cell proliferation. SD treatment also enhances cellular level of tumor suppressor protein 53 (p53) and stimulates cleavage of the nuclear poly (ADP-ribose) polymerase (cleaved-PARP) and also Caspase-3, -8, -9. SD treatment stimulates enzymatic activities of Caspase-3, -8 and -9. All these findings, in addition to the observation that SD inhibits cell viability, suggest that SD inhibits melanoma cell proliferation by arresting the cell-division cycle in a Go quiescent phase and also activates the programmed cell death (apoptosis) via extrinsic and intrinsic pathways. Finally, these studies demonstrate that SD, in a relatively low concentration, displays a very promising inhibiting effect on melanoma B_16_F_10_ tumor cells.

## 2. Results

### 2.1. The Effect of Cell Density on Cell Viability

**Figure 2:**
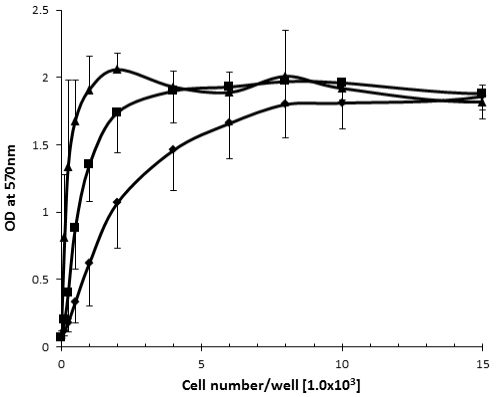
The effect of the increasing cell concentration on viability of the B_16_F_10_ melanoma cells by MTT assay during 24 h (rombus), 48 h (squares) and 72 h (triangles) incubation time with a starting concentration of 100 cells/0.2 mL well.

Prior to conducting studies on the effects of SD on melanoma cell proliferation, we determined the effect of cell density on melanoma cell viability to optimize cell concentration for further assays, and to minimize negative effects of nutrients shortage on cell growth during extended (72 h) cell cultures. Variable concentrations of melanoma cells (from 1 × 10^2^/0.2 mL well to 1.5 × 10^4^/0.2 mL well) were placed into 96 wells plate and incubated at 37 °C in standard conditions. After 24 h, 48 h and 72 h the cell viability was determined by MTT assay. As illustrated in Figure 2 the relative activities of mitochondrial reductase increase as the concentrations of the cells increase until reaching a plateau. In the case of 24 h incubation time, the plateau was observed at ~6 × 10^3^ seeded cells/0.2 mL media. For 48 h of incubation the plateau was observed at ~2 × 10^3^ seeded cells/0.2 mL media, whereas for 72 h the plateau was observed at ~1 × 10^3^ seeded cell/0.2 mL media. 

### 2.2. SD Reduces Viability of the B_16_F_10_ Melanoma Cells

**Figure 3:**
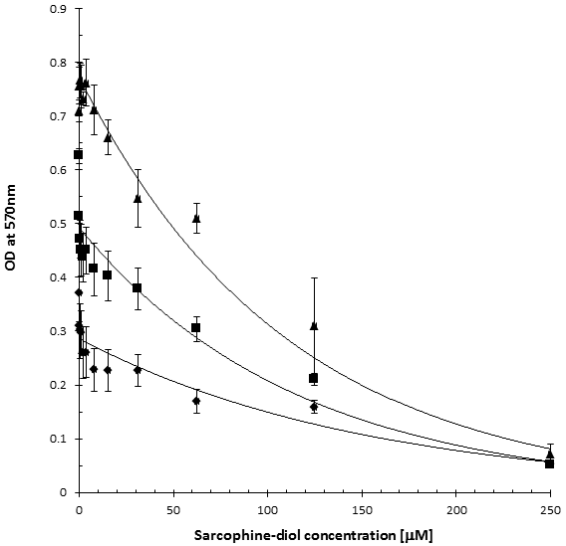
The effect of sarcodiol (SD) on viability of the F_16_B_10_ melanoma cells by MTT assay after 24 h (rombus), 48 h (square) and 72 h (triangle).

The effects of the increasing SD concentrations on melanoma cells viability were determined. Cells were seeded into 96 wells plate at a concentration of 3 × 10^2^/0.2 mL well and incubated for 24 h at 37 °C under standard conditions. Old media was replaced by fresh media containing an increasing concentrations of SD (ranging from 0 µM to 250 µM final concentrations), and cells were incubated for another 24 h, 48 h and 72 h, respectively. The viability of cells was determined by MTT assay. As illustrated in Figure 3, addition of increasing concentrations of SD (ranging from 0 µM to 250 µM) produces a concentration-dependent decrease in formation of the purple formazan crystals by cells. A maximum of ~90–95% inhibition of cell viability was observed at a 250 µM concentration of SD, and these values are valid for both short and longer exposure of cell to SD. The IC_50_ value was estimated at ~70–80 µM concentration of SD and this value is valid for 24 h, 48 h and 72 h incubation times. It is worth mentioning that SD treatment for 72 h at 800 µM inhibited 44% of cell viability in monkey kidney CV-1 cells [3] compared to 80 µM that inhibited 50% of melanoma B_16_F_10_ cells which suggest that SD exhibits much less cytotoxicity in normal cells that in melanoma cells.

### 2.3. SD Inhibits *de Novo* DNA Synthesis

**Figure 4:**
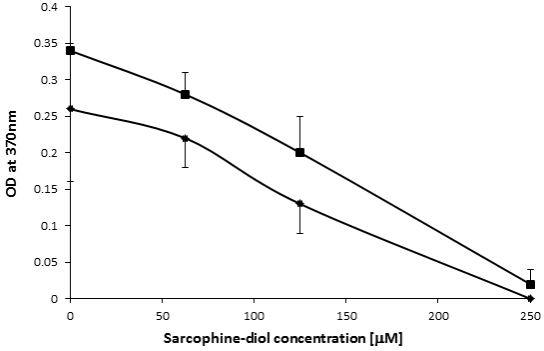
The effect of sarcodiol (SD) on DNA synthesis in B_16_F_10_ melanoma cells after 24 h (rombus) and 72 h (squares) using the BrdU cell proliferation assay.

To better understand the inhibitory effect of SD on cell viability, we examined whether SD treatment affects DNA synthesis. Cell concentrations ranging from 5 × 10^2^/0.2 mL well and 2 × 10^3^/0.2 mL well were seeded into 96 well plates and incubated under standard conditions for 24 h to allow cells attachment to the base. Old media was then replaced with fresh media containing increasing concentrations of SD (ranging from 0 µM to 500 µM final concentrations), and incubated for another 24 h and 72 h, respectively. As illustrated in Figure 4, SD inhibits the incorporation of BrdU into *de novo* synthesized DNA in a dose-dependent manner regardless of the time of treatment. Maximum inhibition was observed at a concentration of 250 µM of SD. IC_50_ value was estimated at ~110 µM of SD. This was valid for both shorter and longer exposure of the cells to SD.

### 2.4. SD Enhances DNA Fragmentation

**Figure 5:**
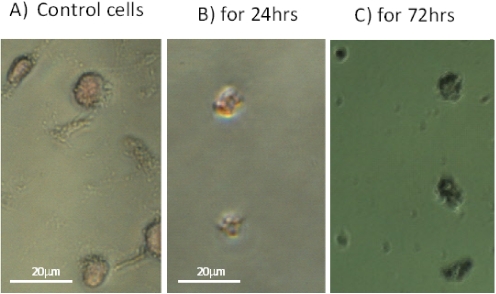
Light microscope images showing the effect of 250 µM concentration of SD on DNA fragmentation in B_16_F_10_ melanoma cells: Image A shows the untreated controls. Images B and C show SD treated cells for 24 h and 72 h, respectively. Note that the untreated control cells of different shapes stains only slightly with pink color, whereas those treated with SD have nearly identical rounded shapes and are stained with deep red color.

We studied the effect of SD on the structural integrity of the DNA molecules in mouse B_16_F_10_ melanoma cells. Cells were seeded at a concentration of 2 × 10^3^/0.2 mL well and incubated at 37 °C for 24 h. The growth media was exchanged with fresh media containing DMSO alone (as a control) and containing 250 µM of SD in DMSO. Cells were incubated at standard conditions for another 24 h and 72 h. The effect of SD on DNA in cells treated with SD compared to the untreated control was visualized using the TACS 2TdT-Blue label apoptosis detection kit together with light microscopy. As shown in Figure 5A, the untreated control cells are characterized by having different shapes and their nucleus is very slightly stained with light pink color. In sharp contrast all the cells treated with SD are characterized by having identical rounded shapes and have nearly the same size (Figure 5B,C). Their nucleus DNA is labeled very strongly with Nuclear Fast Red dye. These data show that SD treatment produces fragmentation in the DNA molecules, and this effect is clearly visible already during first 24 h of SD treatment. 

### 2.5. Effect of SD on the Total Protein Contents in Melanoma Cells

In order to determine whether differences in the rates of *de novo* DNA synthesis and DNA fragmentation in melanoma cells resulting from treatment with SD can affect the total cellular protein content, we developed techniques to thoroughly extract proteins from melanoma cells The control cells were seeded at a concentration of 4 × 10^4^ cells/10 mL of media, and 1 × 10^4^ cells/10 mL of media in 40 mL flasks and incubated for 24 h. Cells treated with SD were seeded in the amounts of 8 × 10^4^/10 mL media and also incubated for 24 h under standard conditions. Media were replaced with new media containing either 0.5% DMSO (control) or 250 µM concentrations of SD in DMSO (test) and cells were incubated for another 24 h and 72 h. At appropriate time cells were harvested by trypsinization, froze in liquid nitrogen, weighed, and used for protein determination. The values for the control experiment in µg protein/mg of frozen tissue are 19.6 ± 0.3 (*n* = 3) and 20.1 ± 0.4 (*n* = 3) for 24 h and 72 h, respectively. The same values for the SD treated cells were 18.3 ± 0.8 (*n* = 3) and 19.1 ± 0.6 (*n* = 3).

### 2.6. SD Inhibits the Levels of Protein Markers for Cell Proliferation

**Figure 6:**
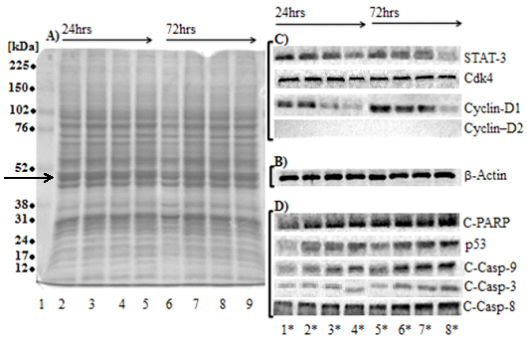
Effect of SD on levels of protein markers for cell proliferation. Panel A: Coomassie-blue stained the 8.5% SDS-PAGE of a total monomer to cross-linker ratio of 29:1 of total protein extracts (20 µg/well) derived from untreated control cells (lanes 2 and 6) and cells treated with 62.5 µM, 125 µM and 250 µM of SD for 24 h (lanes 3–5) and 72 h (lanes 7–9), respectively. Panel B: Western blot of β-actin stained with antibody. (Lane 1) molecular mass standards (in kDa), from top to bottom, are 225, 150, 102, 76, 52, 38, 31, 24, 17 and 12 (Arrow indicates position of actin). Panel C: Western blots of STAT-3, Cdk4 and cyclin-D1 and D-2 stained with their respective antibodies. Panel D: Western blot of the cleaved-PARP, p53, cleaved-Caspases-3, -8 and -9 stained with their respective antibodies. Lanes 1*–4* represent treatment with 0 µM, 62 µM, 125 µM and 250 µM concentrations of SD for 24 h, lanes 5*–8* represent treatment with 0 µM, 62.5 µM, 125 µM and 250 µM concentrations of SD for 72 h.

Figure 6A, shows protein patterns of total protein extracts from untreated control melanoma cells and cells treated with SD on Coomassie-blue stained SDS-PAGE. Position of actin on the 8.5% SDS-PAGE stained with Coomassie-blue is indicated by an arrow. When transferred to nitrocellulose membrane, probing with antibody and visualized by ELC reagents the actin molecule is represented by a single, sharp band (Figure 6, panel B). This proves that our protocol for protein extraction inhibits efficiently proteolysis of actin, a cytoskeleton protein that is easily assessable for proteolytic degradation. 

Cellular levels of protein markers for cell proliferation were measured using Western blotting analysis in cells treated with the increasing concentrations of SD (from 0 µM to 250 µM) for 24 h and 72 h compared to untreated controls. These markers are: STAT-3 and Cdk4 and its low molecular mass activators, cyclin-D1 and -D2.

Since STAT-3 is known to be expressed in large quantities in different human tumors and cell line [16,17], we investigated the cellular expression levels of STAT-3 in melanoma cells exposed to various concentrations of SD. As illustrated in Figure 5B, SD treatment inhibits expression of the STAT-3 protein in a dose-dependent manner during 24 h and 72 h exposures. Maximal inhibition (60–70% inhibition) was occurred at a concentration of 250 µM concentrations of SD. 125 µM concentrations of SD inhibit expression levels of STAT-3 to a lesser extent (~50% inhibition), whereas 62.5 µM concentrations of SD produced a minor inhibition. 

High activity of the Cdk4-cyclin-D protein complex is restricted to the G1-S phase. Figure 5C shows that SD treatment does not affect cellular level of Cdk4 protein compared to the untreated controls at all concentrations used at either 24 h or 72 h exposure (Table 1). However, SD treatment has changed the cellular levels of cyclin-D1 (an activator of Cdk4 kinase) (Figure 6C). The lowest expression levels of cyclin-D1—barely detected in cells treated with 250 µM concentrations of SD both for 24 h and for 72 h (Table 1). The cellular levels of cyclin-D2 were not detectable in all cell extracts.

**Table 1:** The effect of the increasing concentrations of SD on the relative contents of protein markers for cell proliferation and apoptosis in the untreated control B_16_F_10_ melanoma cells, and cells treated with 62.5, 125 and 250 µM concentrations of SD for 24 h and 72 h. Values are means ± SDV (standard deviation); *n* = no. of determinations and (*) values with statistical difference (*p* < 0.05) as compared to untreated control cells.

		Sarcophine-diol concentration (µM)							
		0	62.5	125	250	0	62.5	125	250
		24 h treatment				72 h treatment			
**Proliferation markers**	STAT-3	100%	78.8 ± 9.5 *n* = 4	62.9 ± 10.4 *n* = 4 (*)	36.0 ± 6.0 *n* = 4 (*)	100%	70.4 ± 18.2 *n* = 3	59.8 ± 8.4 *n* = 3 (*)	41.8 ± 10.5 *n* = 3 (*)
	Cdk4	100%	105.3 ± 9.2 *n* = 3	92.8 ± 14.3 *n* = 3	101.8 ± 15.3 *n* = 3	100%	112.5 ± 27.2 *n* = 3	97.4 ± 18.9 *n* = 3	118.7 ± 20.1 *n* = 3
	Cyclin-D1	100%	75.3 ± 10.5 *n* = 3	35.5 ± 7.2 *n* = 3 (*)	7.7 ± 14.2 *n* = 3 (*)	100%	87.8 ± 12.6 *n* = 3	48.9 ± 9.8 *n* = 3 (*)	4.9 ± 6.2 *n* = 3 (*)
	Cyclin-D2	ND	ND	ND	ND	ND	ND	ND	ND
**Apoptosis markers**	C-PARP	100%	123.2 ± 18.5 *n* = 3	132.1 ± 18.1 *n* = 3 (*)	168.3 ± 24.1 *n* = 3 (*)	100%	189.3 ± 32.2 *n* = 3 (*)	216.0 ± 42.7 *n* = 3 (*)	276.0 ± 56.8 *n* = 3 (*)
	p53	100%	128.0 ± 23.9 *n* = 3	132.1 ± 19.6 *n* = 3 (*)	132.7 ± 16.9 *n* = 3 (*)	100%	141.5 ± 33.3 *n* = 3	159.9 ± 22.6 *n* = 3 (*)	177.6 ± 19.4 *n* = 3 (*)
	C-Casp-9	100%	124.3 ± 27.7 *n* = 3	132.3 ± 20.5 *n* = 3 (*)	147.4 ± 18.1 *n* = 3 (*)	100%	130.6 ± 23.5 *n* = 3	158.0 ± 31.6 *n* = 3 (*)	179.1 ± 18.9 *n* = 3 (*)
	C-Casp-3	100%	120.2 ± 14.3 *n* = 3	133.3 ± 9.9 *n* = 3 (*)	140.3 ± 16.7 *n* = 3 (*)	100%	128.2 ± 25.5 *n* = 3	141.7 ± 22.5 *n* = 3 (*)	174.2 ± 21.0 *n* = 3 (*)
	C-Casp-8	100%	119.5 ± 22.6 *n* = 3	132.3 ± 25.3 *n* = 3 (*)	160.3 ± 18.7 *n* = 3 (*)	100%	123.9 ± 25.1 *n* = 3	178.3 ± 33.6 *n* = 3 (*)	213 ± 51.2 *n* = 3 (*)

### 2.7. SD Increases the Expression Levels of Protein Markers for Apoptosis

Over-expression of cysteine proteases which inactivate poly-(ADP-ribose)polymerase (PARP)—an enzyme involved in DNA repair—is a hallmark of apoptosis, a process that commits cells to die [18,19]. We studied the effect of SD on the expression levels of the cleaved-PARP (an inactive form of polymerase) in melanoma cells. Treatment of melanoma cells with SD (in concentrations ranging from 0 µM to 250 µM) increases cellular levels of the cleaved-PARP in a dose-dependent manner compared to untreated controls (Figure 5D). The highest expression levels of inactive PARP (roughly 2.8-fold higher) was observed at 250 µM concentration of SD for 72 h. 1.7-fold higher content of inactive PARP was detected in cells exposed to the same high concentration of SD for 24 h. Treatment with 63 µM and 125 µM concentrations of SD for 24 h produces only slight increase in the cellular levels of cleaved-PARP.

Because p53 protein is a tumor suppressor protein [20,21,22], we studied the effect of SD on the cellular level of this protein. SD increases the expression levels of p53 in a dose-dependent manner (Figure 5D). The highest expression level of p53 (roughly ~1.3-fold and ~1.8-fold increase compared to untreated controls) was observed at 250 µM concentrations of SD for 24 h and 72 h (Table 1). Less levels of p53 were observed in cells treated with SD concentrations of 62.5 µM and 125 µM for 72 h.

Caspases are the cysteine proteases that are synthesized as inactive precursors and activated by enzymatic cleavage during programmed cell death [18,19]. Because cleaved-Caspase-3 can be considered as biomarkers for apoptosis, we studied the effect of SD on activating the caspase’s pathway(s). As illustrated in Figure 5D, SD increases the cellular level of cleaved-Caspase-3 in the B_16_F_10_ melanoma cells in a dose-dependent manner. Highest expression levels of cleaved-Caspase-3 was observed in cells treated with 250 µM concentrations of SD, for both 24 h and 72 h exposure. Less levels of cleaved-Caspase-3 (around 50–70%) were observed in cells treated with 125 µM and 62.5 µM concentrations of SD (Table 1).

As illustrated in Figure 6D and summarized in Table 1, SD enhances cleavage of Caspase-8 and Caspase-9 in a dose-dependent manner. The highest levels of these proteins (roughly 2.1-fold and ~1.8-fold compared to untreated controls) were observed in cells treated with 250 µM concentrations of SD for 72 h. Smaller values were observed in cells treated with 250 µM concentrations of SD for 24 h although the cellular levels of active Caspase-8 and -9 in these cells are ~1.6-fold higher and ~1.5-fold higher than in the untreated controls. Treatment with 62.5 µM concentrations of SD produces only slight (roughly 1.3-fold) increase in levels of cleaved Caspase-8 and -9 in both 24 h and 72 h exposures.

### 2.8. SD Increases Enzymatic Activities of the Cleaved-Caspases-3, -8 and -9

**Figure 7:**
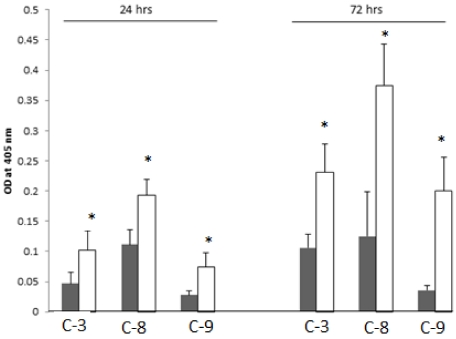
The effect of SD on the enzymatic activities of the cleaved-Caspase-3, -8 and -9, respectively, in the B_16_F_10_ melanoma cell protein extracts for 24 h and 72 h. Each bar represents mean ± SDV of 3 independent determinations with 3 estimates each, and (*) values with statistical difference (*p* < 0.05) as compared to untreated control cells.

We studied the enzymatic activities of cleaved-Caspase-3, -8 and -9 in protein extracts from cells exposed to SD and untreated controls. Cells at a concentration of 6 × 10^5^/75 mL of growth media were incubated under standard culture conditions for 24 h. Then media was exchanged with a fresh media containing either 0.5% DMSO or 250 µM concentrations of SD dissolved in DMSO. Control cells were incubated for 24 h, whereas cells treated with SD were incubated for 24 h and 72 h. At the end of treatment, cells were harvested and lysed. Protein extracts were prepared for determination of enzymatic activities of the cleaved-Caspase-3, -8 and -9. As illustrated in Figure 7, protein extract from cells treated with SD for 24 h exhibited ~2.0-fold higher activity of caspase-3 compared to control cells. Nearly the same increase in activity of cleaved-Caspase-3 was observed in cells exposed to 250 µM SD for 72 h as compared to controls.

Similar pattern in enzymatic activities was observed for cleaved-Caspases-8 and-9. Specifically, protein extract from cells treated with SD for 24 h showed ~1.8-fold higher and 2.7-fold higher activity of cleaved-Caspase-8 and-9, as compared to control values. As also illustrated in Figure 6, higher difference in the activities of cleaved-Caspase-8 and -9 (~roughly 3-fold increase and 6.0-fold increase) were observed in cells exposed to SD for 72 h as compared to control cells.

### 2.9. Discussion

Cell viability can be determined by measuring the mitochondrial metabolic activity. The enzymatic-based assay which measures reduction of soluble tetrazolium salts to insoluble formazans by mitochondrial reductase has been widely used to assess potency of chemotherapeutic anticancer drugs [23,24]. As this is the first time to determine the anti-cancer activity of SD against melanoma cells, we conducted the MTT assay to determine the effects of SD on viability of melanoma B_16_F_10_ cells. We demonstrated that SD treatment induces a concentration-dependent inhibition of mitochondrial metabolic activity in our melanoma cells. It is worth mentioning that these values are valid for 24 h, 48 h and 72 h exposure of melanoma cells to SD. The next step was to investigate the inhibitory effects of SD on melanoma cell proliferation. At first, we investigated the effect of SD treatment on DNA synthesis. Cell proliferation requires replication of cellular DNA content and the monitoring of DNA synthesis is more direct parameter for cell proliferation as compared to mitochondrial metabolic activity. Using Elisa colorimetric-based assay we investigated the incorporation of BrdU into the DNA molecule. We found that SD inhibits the rates of BrdU incorporation onto DNA during its *de novo* synthesis. Our data also show that the inhibitory effect of SD on DNA synthesis depends on SD concentrations. SD at a concentration of 250 µM completely inhibits DNA synthesis. To investigate the possible mechanism(s) by which SD inhibits the incorporation of BrdU onto *de novo* synthetized the DNA molecule, we studied the expression levels of p53 and STAT-3 proteins cells treated with SD as compared with untreated control. 

The p53 protein, is a tumor suppressor protein, and commonly accepted as an important indicator for inducing apoptosis [20,21]. p53 protein was demonstrated to activate transcription of many pro-apoptotic factors such as BAX, Bak, Fas/APO-1, PIDD, *etc.* It was also found to suppress the transcription of many anti-apoptotic genes. These suppressed genes include Bcl-2, Bcl-X_L_, *etc.* [25,26]. In addition, tumor suppressor protein itself shows capability for up-regulating apoptosis, without transcription, by directly localizing to mitochondria following the DNA damage and interacting with anti-apoptotic proteins to free pro-apoptotic proteins such as BAX [20,25,26,27]. It also was demonstrated that under stress conditions, p53 can contribute to apoptosis by facilitating transport of death receptors such as Fas/APO-1 and/or Killer/DR5 from cytoplasmic stores to the cell surface as required for programmed cell death [26,28]. In these studies, we did not investigate a molecular mechanism(s) of SD stimulated apoptosis via p53 signaling pathway(s). Based on studies by Boni *et al*. [29], it is unlikely that high cellular content of p53 in melanoma cells could override the activities of many Calcium binding proteins that are involved in cell proliferation. One of those calcium binding proteins, 21 KDa S100B, was found to have an ability to directly interact with the p53 tumor suppressor protein in melanoma cells [30,31]. It was found that p53 inhibits the pro-apoptotic functions of this protein [31,32,33].

STAT-3 belongs to the protein family of cytoplasmic transcription factors that have been identified to play a role in genes expression that are involved in cell survival, proliferation, chemo-resistance, and angiogenesis [34,35]. Activation of STAT-3 occurs through phosphorylation of its critical tyrosine and serine residues and occurs in response to cytokine and growth factor receptor signaling [36,37]. Once activated, STAT-3 translocates from the cytoplasm into the nucleus and binds to specific areas of DNA. STAT-3 is known to exist in a high concentration in different human tumors and tumor cell lines [38,39,40], therefore it becomes an attractive target for cancer therapy [16,17]. Several studies demonstrated that inhibition of STAT-3 activation, as regards to both phosphorylation and expression, correlates with suppression of human malignant cells in experimental systems both in the *in vitro* and *in vivo* conditions [16,17,40]. We studied the effect of SD on total protein expression level of STAT-3 in melanoma cells, and found that it decreases the content of STAT-3 in B_16_F_10_ melanoma cells, and this observation is in agreement with findings by others that inhibition of cell proliferation characterizes in lower cellular level of STAT-3 [40]. Owing to the fact that the anti-STAT-3 antibody that we used recognizes both phosphorylated and unphosphorylated isoforms of STAT-3, it is very likely that SD also inhibits phosphorylation of STAT-3 protein, and this process could additionally inhibit pro-proliferation activity of STAT-3 in melanoma cells.

To better understand the inhibitory effect of SD on melanoma cell proliferation, we were interested in whether SD treatment could also stimulate DNA fragmentation. Using a TACS2TdT-Blue Label *in situ* kit, we tested this hypothesis. We compared the extent of DNA fragmentation in control melanoma cells with identical cells treated with 250 µM SD for 24 h and 72 h. As illustrated in Figure 4A–C, SD enhances DNA fragmentation, and this effect seems to be identical regardless time of cells exposure to SD whether it was 24 h or 72 h. Labeling of DNA by Nuclear Fast Red is completely different in control untreated cells than those exposed to SD. While untreated cells of the irregular shapes are labeled only slightly with pink dye, cells treated with SD that characterize in having quite identical rounded shapes have nucleus strongly stained with deep red color. 

Since degradation of DNA occurs in the quiescent Go state, we were also interested in whether SD affects the cell-division cycle. It is commonly accepted that upon entering the quiescent state, cells do not divide, and our studies with the use of MTT show that SD inhibits B_16_F_10_ melanoma cell proliferation. In view of these findings, we determined the expression level of Cdk4 kinase and its activators (cyclin-D1 and cyclin-D2) in control cells and cells treated with SD. Cdk4, a member of cyclin-dependent serine/threonine protein kinases family is positively expressed in cells, whereas cyclins, their activators are synthesized at the specific stages of the cell-division cycle, in response to various molecular signals. Once it is associated, the cyclin-CDK protein complex moves from cytosol to the nucleus [41,42] and becomes involved in the activation of target protein(s) via phosphorylation in order to orchestrate coordinated entry of the cell into the next phase of the cell-division cycle. High activity of the complexes between Cdk4 and the D-type cyclins is restricted to the G1-S phase that is characterizes by cell size increase, preparation for DNA synthesis and, finally, DNA replication [43,44]. While in agreement with findings by other investigators [45] our data show that SD does not affect the cellular level of Cdk4, and this is valid for both longer and shorter exposures of the cells to SD. Interestingly, SD in a concentration dependent-manner inhibits expression level of cyclin-D1. This latter observation suggests that SD inhibits entry of cells into the G1-S phase, arresting them in the quiescent Go state. While also in agreement with findings by other investigators [45] our data show a lack of expression of cyclin-D2 in melanoma B_16_F_10_ cells.

The latter two findings raise the question of whether the content of cleaved-PARP is different in the control cells *versus* SD-treated cells. The intact PARP molecule is a 116 kDa nuclear poly-(ADP ribose) polymerase that appears to be involved in DNA repair in response to environmental stress [46] and helps cells to maintain their viability. Inactivation of PARP can be facilitated by many ICE-like caspases *in vitro* [47], and it is one of the main cleavage targets for Caspase-3 *in situ*, the key executioners of apoptosis [48]. In human PARP, cleavage occurs between Asp^214^ and Gly^215^, separating the NH_2_-terminal DNA binding domain of molecular mass of 24 kDa from the 89kDa COOH-half fragment [47]. The 89 kDa fragment of PARP contains catalytic and an auto-modification domains. The latter domain is responsible for dissociation of the intact PARP molecule from the DNA after repairing the damaged DNA. The NH_2_-terminal fragment of PARP facilitates a permanent interaction of PARP with DNA. Since the interaction between the NH_2_-terminal domain of PARP with the DNA molecule inhibits binding of the native PARP to DNA, and since this binding reduces PARP’s ability for repairing the damaged DNA [49], it is very likely that this mechanism enables the SD treated cells to repair the damaged DNA, and leads to apoptosis [49]. Our data showing that treatment with SD enhances cellular level of the 89 kDa COOH-half fragment of PARP are in agreement with this hypothesis. 

It is commonly accepted that active Caspase-3 is capable of inactivating PARP via PARP cleavage into the COOH-half and the NH_2_-terminal DNA-binding domain [19,50]. Proteolytic enzymes of the Caspase family are a hallmark for apoptotic cell death [51,52]. Cleaved-Caspase-3 is a final executioner of classic apoptotic pathway. Two processes have been identified to activate caspase-3. In some forms of apoptosis, the extrinsic apoptotic pathway that is initiated by activation of the apical Caspase-8 following death receptor ligation directly activates Caspase-3 [52]. Cleaved-Caspase-8 is an active form of Caspase-8. Our data shows that both protein expression levels and enzymatic activity of cleaved-Caspase-8 are higher in cells treated with SD as compared to those untreated controls suggesting that SD treatment activates Caspase-8 cascade. In another form of apoptosis, cellular stress leads to activation of the intrinsic apoptotic pathway initiated by the apical Caspase-9 [53] that also remains under control of Caspase-8. In this route of apoptosis cleaved-Caspase-9 activates caspase-3 [54,55]. Our data show that SD treatment also activates the Caspase-9 apoptotic pathway. Certainly, more studies are needed to explore the molecular mechanism(s) of SD inhibition of cell proliferation and activation of apoptosis in melanoma cells.

## 3. Experimental Section

### 3.1. Materials and Reagents

The gel apparatus and material for electrophoresis were purchased from Hoefer (San Francisco, CA, USA) and Bio-Rad (Richmond, CA, USA). The nitrocellulose transfer membranes having a pore size of 0.22 µm in diameter was purchased from Santa Cruz Biotechnology, Inc. (Santa Cruz, CA, USA). All the commonly used reagents, mouse monoclonal anti-p53 antibodies, 10,000 units of Penicillin and 10 mg Streptomycin stock solution and Trypsin-EDTA 10X-solution were purchased from Sigma-Aldrich (St. Louis, MO, USA). Mouse monoclonal antibodies against cleaved-PARP, cleaved-Caspase-8 and rabbit monoclonal antibodies against cleaved-Caspase-3, and cleaved-Caspase-9 were purchased from Cell Signaling (Danvers, MA, USA). Mouse monoclonal antibodies against β-actin, Cdk4 and STAT-3, rabbit antibodies against cycline D1 and D2, goat anti-mouse and goat anti-rabbit both horseradish-conjugated (HRP) secondary antibodies were purchased from Santa Cruz Biotechnology, Inc. (Santa Cruz, CA, USA). The B_16_F_10_ mouse melanoma cell line, Dulbecco’s Modified Eagle’s Media (DMEM) supplemented with L-glutamine and Fetal Bovine Serum (FBS) were purchased from the American Type Culture Collection (ATCC) (Manassas, VA, USA). 

### 3.2. Synthesis of SD

Sarcophine was isolated from the soft coral *Sarcophyton glaucum* using the reported procedure [10,14], Sarcodiol (SD) was synthesized following our reported procedure [3]. SD was dissolved in 100% dimethyl sulfoxide (DMSO) to prepare 50 mM stock solution. The 50 mM stock solution of SD was further diluted in DMSO to obtain the appropriate concentrations. In all assays the final concentration of DMSO in growth media was 0.5%, whereas the final concentrations of SD vary from 0 µM to 250 µM. 

### 3.3. Cell Culture

Mouse melanoma B_16_F_10_ cell line was grown in DMEM media supplemented with 10% FBS and a 1% solution containing 100 U/mL penicillin and 100 mg/mL streptomycin to inhibit bacteria growth. Cells were grown in 37 °C in a humidified atmosphere containing 5% CO_2_ and 95% air. 

### 3.4. Total Cellular Protein Extract

Cells were detached from the surface of culture flasks by short exposure to a minimal amount of trypsin. Immediately after detachment, the cell suspension was diluted with 10-fold excess of growth media to inhibit proteolytic activity of trypsin. Upon transfer to the centrifuge tubes, cells were pelleted by centrifugation in 25 °C at 1700 rpm for 5 min. The supernatant was discarded whereas the cell pellet was thoroughly washed with phosphate buffered saline (PBS) to remove protein contamination from FBS. The washing procedure was repeated 3 times. Cells were then solubilized in 3–5 volume of an ice-cold extraction buffer by passing several times through a pipet tip. Cell lysate was then kept in an ice-cold extraction buffer for 10–15 min and then spin down in 4 °C at 14,000 rpm for 20 min. The supernatant was collected and stored at −20 °C for determination of protein content and Western blot analyses. 

For routine assays, the extraction buffer contained: 20 mM Tris(hydroxymethyl)aminomethane (Tris)-HCl, pH 7.8; 150 mM NaCl; 1% octylphenoxypolyethoxyethanol (Triton X-100); 1% sodium deoxycholate; 0.1% sodium dodecyl sulfate (SDS); 2 mM ethylenediaminetetraacetic acid (EDTA), 2 mM ethylenebis(oxyethylenenitrilo)tetraacetci acid (EGTA), 2 mM sodium azide (NaN_3_), 2 mM dithiothreitol (DTT). To minimize proteolytic degradation during extraction, we supplemented the extraction buffer with a protease inhibitor cocktail (Roche Diagnostic GmbH, Mannheim, Germany) according to the manufacturer’s protocol (one tablet of protease inhibitors was diluted in 1 mL of double distilled water and added to 7 mL of extraction buffer prior to use).

### 3.5. Determination of Protein Content

Total protein content in the cell extract was determined colorimetrically using a detergent compatible bicinchoninic acid (BCA) protein assay kit according the manufacturer’s protocol (Pierce, BCA, Rockford, IL, USA), using bovine serum albumin (BSA) as a standard.

### 3.6. Western Blot Analyses of Protein Markers for Cell Proliferation and Apoptosis

The total cell protein extract in the amounts of ~15–20 μg protein/well was resolved the 8.5% SDS-PAGE, using a total monomer to cross-linker ratio of 29:1. Once separated, proteins were transferred in electric field onto the nitrocellulose membrane. Transferred proteins were identified following this procedure: The nonspecific binding sites were blocked by Tris-buffered saline, pH 7.5 supplemented with 0.05% polyoxyethylenesorbitan monolaurate (Tween 20) and 5% non-fat milk at room temperature for 1 h. Membranes were then probed with primary antibodies at room temperature for 1.5 h using the a dilutions of 1:1000 for β-actin, cleaved-PARP, cleaved-Caspase-3, -8, -9, Cdk4 and cyclin-D1 and -D2, and a dilution of 1:500 for STAT-3. Primary antibodies were diluted in Tris-buffer saline, pH 7.5 containing 0.05% Tween 20 and 1% non-fat milk. After removal of unbound antibodies, membranes were thoroughly washed with Tris-buffer saline, pH 7.5 containing 0.05% Tween 20. Secondary antibodies were then applied. All secondary antibodies were diluted in Tris-buffer saline, pH 7.5 supplemented with 0.05% Tween 20, using a dilution of 1:2000 for detection of β-actin, cleaved-PARP, cleaved Caspase-3, -8, -9, Cdk4 and cyclin-D1 and -D2 and a dilution of 1:1000 for probing the STAT-3 protein. Membranes were exposed to secondary antibodies at room temperature for 1 h. Then membranes were thoroughly washed with Tris-buffer saline, pH 7.5 to remove unbound antibodies. Protein bands were visualized by Western Blotting Luminol Reagent (Santa Cruz Biotechnology, Inc., Santa Cruz, CA, USA) and then quantified using UVP Biochem Gel Documentation System (UVP, Inc., Upland, CA, USA). 

### 3.7. Cell Viability Assays

#### 3.7.1. Staining of the Cells with Trypan-Blue

For the purpose of preparing cell suspension having defined amounts of living cells, a quick Trypan-blue exclusion staining method was used. Briefly, cell suspension in growth media was diluted twice with a 0.4% Trypan-blue solution (AMRESCO, Solon, OH, USA). Concentrations of living cells were determined with the use of Cellometer Auto T4 Plus Cell Counter (Nexcelom Bioscience LLC, Lawrence, MA, USA) equipped with Cellometer Automated Cell Counts software.

#### 3.7.2. MTT Assay

Cell viability was also determined by the Elisa-type MTT kit following the manufacturer’s protocol (Biotium, Inc., Hayward, CA, USA). This colorimetric assay is based on reduction of the yellow tetrazolium salt to a purple formazan crystal by mitochondrial reductase which is active in living cells. This method was used for studying the effects of SD on melanoma cell viability. We performed MTT assay in a 96 well plate. 10 μL of MTT solution/0.1 mL media was added to each well. The reaction mixture was then incubated at 37 °C for 4 h. 200 μL of DMSO was added to dissolve the purple formazan crystals. Color absorbance of samples was measured at 570 nm with a reference wavelength of 630 nm to correct background for the blank sample (growth media without cells) using a Type M2 Spectra Max Microplate Reader (Sunnyvale, CA, USA) equipped with SoftMax PRO4.8 software. 

### 3.8. Determination of DNA Synthesis

The rates of DNA synthesis were determined by measuring BrdU incorporation into *de novo* synthetized DNA and were carried out using the BrdU Elisa-type kit according to the manufacture’s protocol (Roche Diagnostics, GmbH, Mannheim, Germany). At the end of incubation period, 10 μL of BrdU labeling solution per 0.1 mL media was added to each well and the reaction mixture was placed at 37 °C for 4 h. Following removal of the medium, 200 μL of FixDenat solution was added and the cells were incubated at room temperature for another 30 min. After emptying the wells, a 100 μL of the anti-BrdU solution was added and the reaction mixture was incubated at room temperature for another 90 min. After removal of the antibodies and after several washes with PBS, 100 μL of the Substrate Solution was added to each well. After incubation at room temperature for 30 min, the color absorbance of the samples was measured at 570 nm with a reference wavelength of 630 nm to correct background for blank sample (growth media without cells) using a Type M2 Spectra Max Microplate Reader (Sunnyvale, CA, USA) equipped with SoftMax PRO4.8 software. 

### 3.9. Determination of DNA Fragmentation *in Situ*

To visualize the effect of SD on DNA fragmentation *in situ* we used TACS 2TdT-Blue Label Detection kit from R & D Systems (Minneapolis, MN) together with a light microscope. We performed this assay using cells cultured in a 96 well plate and exposed to 0.5% DMSO alone and a 250 μM concentration of SD for 24 h and 72 h. At appropriate time, cells were quickly rinsed with PBS buffer. Then, 3.7% formaldehyde in PBS buffer was added and the cells were fixed at room temperature for 10 min and thoroughly washed with PBS buffer to remove formaldehyde. Cell membranes were then permeabilized with Proteinase K Solution at room temperature for 20 min, and then washed with distilled water. After a treatment with Quenching Solution for 5 min and washing with PBS, cells were treated with the TdT Labeling Buffer at room temperature for 5 min. Cells were then incubated in the Labeling Reaction Mix at 37 °C for 1 h in humidifying chamber. Cells were immersed in the TdT Stop Buffer Solution at room temperature for 5 min. After washing with PBS, the Stop-HRP Solution was applied for 10 min. Following several washes with PBS, the cells were immerse in TACS-Blue Label Solution for 3 min, then washed for a few times with deionized water and counterstained with Nuclear Fast Red for 1 min. After several washes with ethanol to remove the excess staining solution, cells were covered with double distilled water and examined under a model AX10 light microscope connected to AxioVision Rel. 4.7 software via camera model AxioCam MRc5 (Zeiss, Germany).

### 3.10. Determination of Enzymatic Activities of the Cleaved-Caspases-3, -8 and -9

Enzymatic activities of Caspase-3, -8 and -9 in the cell extract were measured using ApoTarget kit, according to the manufacturer’s protocol (Invitrogen Corp., Camarillo, CA, USA). At the end of experiment, cells (test and control) were harvested by trypsinization, washed thoroughly with ice-cold PBS buffer and lysed using a chilled cell lysis buffer provided by the manufacturer for 10 min. Proteins were collected by centrifugation at 10,000 rpm for 5 min. Protein concentration in each supernatant fraction was diluted to the same concentration. Each sample containing 100 μg of protein in 50 μL of lysis buffer was added to reaction buffer containing 200 μM of Caspase substrate, which is a synthetic peptide conjugated to a chromophore p-anilide (pNA). For determination of the cleaved-Caspase-3 activity, DVED-pNA was used. For the cleaved-Casspase-8, LETD-pNA was used and for cleaved-Caspase-9, LEHD-pNA was used. The reaction mixtures were then incubated at 37 °C for 4 h. Following cleavage, light absorption by the released pNA was measured at 400 nm using a Type M2 Spectra Max Microplate Reader (Sunnyvale, CA, USA) equipped with SoftMax PRO4.8 software. The rates of caspase activities in control cells and cells treated with SD were calculated after subtraction of background absorbance. 

### 3.11. Statistical Analysis

All values are expressed as means ± SDV. A Student’s *t*-test was used for statistical analysis, and a confidence level of *P* < 0.05 was chosen as indication of statistical difference.
